# Scientific Items

**Published:** 1879-01-20

**Authors:** 


### SCIENTIFIC ITEMS.

THE ST. BENOIT TWINS.—These remarkable phenomena were recently taken from their home in St. Benoit, Canada, to Philadelphia, and examined in the presence of hundreds of medical students and doctors by Prof. Pancoast, the most accomplished living expert in teratology—as the science of this kind of monstrosity is called. Though similar anomalies are on record, this is the only known case where so remarkable a human structure has been born alive; and there seems every prospect of the twins living to grow up. Rose and Mary are the names of this wonderfully close-joined pair of sisters that can never know separation. Rose-Mary would be the name for them taken as one. A toy was handed Mary; she smiled, took it and played with it. Rose reached out her baby-hand, got the toy and also smiled and chuckled. The children are separate down to the edge of the ribs, with separate heads, separate arms, two to each, and separate hearts. At the breastbone the bodies are fairly united, but the rest of the organs are common to both. There is but one navel, and the buttocks are united, teriminating on each side with a well formed leg, one for each of the joined twins. Rose's leg is a very little fatter than the one belonging to Mary, but both are of equal length, and perfectly formed. While the head, arms and thorax, with its contents, are separate, it is probable that the intestinal canal is common to both, as there was but one anus, and that, in all probability, the bladder and womb are more or less united, as they have but one common genitalia.

IT is now known that if oils are mixed with sawdust, text-le materials, &c., so as to be exposed to the atmostphere in thin films, oxygen is rapidly absorbed, the temperature of the mass rises, and, under favors able circumstances, actual ignition may ensue. The effect varie- greatly with the kind of oil used, some oils being exceedingly dangerous, while others may be regarded as practically safe; hence, the risk of fire in a factory must be influenced, to a very considerable degree, by the kind of oil used. Olive oil has been considered the least dangerous—mineral oils, pine, linseed, and rape, and their mixture, the most so—while all others are supposed to hold an intermediate position. But according to some recent experiments made abroad, olive oil is to be regarded really among the most dangerous of all, while the hydrocarbons, such as the mineral oils are incapable of producing spontaneous combustion.—*Hehrew Leadae.*

TELEMACHON.—The telemachon is a dynamo-electric machine, an invention by Mr. Wallace, by which electricity generated by water or steam-power is made available for transmitting the power to a distant point through conducting wires. Edison has seized upon the idea as a means of generating electric light, and says: "With fifteen or twenty of these dynamo-electric machines I can light the entire lower part of New York city, using a five-hundred horse-power steam engine." The means that brings the light, can also furnish power and heat, which can also be variously utilized for running sewing machines, warming apartments, cooking, etc. What next?

URANINE.—This is the most recently discovered and perhaps the most remarkable of all the coal tar or aniline group of coloring substances, now so extensively used for the adornment of the finest fabrics. Uranine is said, by chemists, to be the most highly fluorescent body known to science. Its coloring power is astonishing; a single grain will impart a marked color to nearly five hundred gallons of water.

A most interesting experiment, which anybody may try, consists in sprinkling a few atoms of uranine upon the surface of the water in a glass tumbler. Each atom immediately sends down through the water what appears to be a bright green rootlet; and the tumbler soon looks as if it were crowded full of beautiful plants. The rootlets now begin to enlarge, spread and combine, until we have a mass of soft green-colored liquid. Viewed by transmitted light, the color changes to a bright golden or amber hue; while a combination of green and gold will be realized, according to the position in which the glass is held. For day or evening experiment nothing can be prettier than these trials of uranine, which are especially entertaining for the young folks. We are indebted for examples of the color to the editors of the *Scientific American*, who are sending out specimens, free of charge, to all their readers. The subscription to the paper is $3.20 for a year, or $1.60 half year; and a better investment for the money could hardly be named.

THE THERMAL DEATH POINT OF SEPTIC ORGANISM.—Some very careful experiments on this subject have lately been rehearsed to the Royal Society by the Rev. W. N. Dallinger. He found septic organisms living after exposure to a heat of 250° Fahr. He says:

"I followed this with four more experiments, separately and successively made. Two of them were at a temperature of 248°, and two at 250° F. In both of the former, at the end of nine or ten hours, the complete organism in full vigor could be seen; and in one of the cases it was discovered still in the condition shown at Plate 2, and watched until the organism had attained the condition indicated in Plate 2.

But in the two latter instances (heated up to 250°) the living form did not appear during the six days following, although repeatedly looked for.

I concluded, therefore, that the temperature of 250° F. was the limit of endurance which the spore of this form could bear by this method of heating."

Boiling water, therefore, would not destroy these germs.—*Lan. & Clin.*

PROF. DOLBEUR'S EXPERIMENTS, says *Les Mondes*, appear to equal anything in Tyndall's illustrations of the same phenomenon—sound waves. He takes a tube of any material, one to two inches in diameter and fourteen or more inches long; on one of the ends he applies a membrane of silk paper, fine ribbon, or gold-beater's skin, and in its centre fixes with gum a piece of looking glass, not above one-eighth of an inch square, with the reflecting side turned outward. When dry, it is turned to the sunlight, the open end of the tube is put in the mouth, holding the other end in such a manner that the rays of light reflected may fall upon a white wall or on a sheet of paper. If the operator then speaks or sings into the tube, the regular motion of the ray of light presents very pleasing and regular designs, differing only according to the intensity of the sound.

